# Supplementary material for: Incidence of cough from acute exposure to fine particulate matter (PM2.5) in Madagascar: A pilot study
Source: PLOS Glob Public Health. 2024 Jul 26;4(7):e0003530. doi: 10.1371/journal.pgph.0003530 (PMC11280240; doi:10.1371/journal.pgph.0003530)

**S1 Fig. Participant wearing the carrying pouch containing the iPhone 6 with the ResApp^TM^ Health application and the MicroPEM^TM^ monitoring devices.**


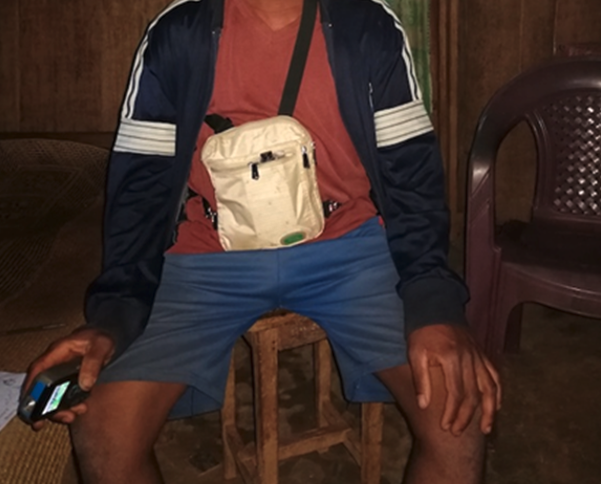

Supplement: S1 Fig — (DOCX) [file pgph.0003530.s001.docx]
