# Supplementary material for: Incidence of cough from acute exposure to fine particulate matter (PM2.5) in Madagascar: A pilot study
Source: PLOS Glob Public Health. 2024 Jul 26;4(7):e0003530. doi: 10.1371/journal.pgph.0003530 (PMC11280240; doi:10.1371/journal.pgph.0003530)

**S2 Fig. Hourly cough recordings for each participant.**

Cough recordings for each participant, identifying hours when 0 coughs per hour were recorded (in red). Sensitivity analyses explored the association between cough and PM 2.5 that excluded data points when 4h+, 6h+, or 8h+ of consecutive hours of recording captured 0 coughs.


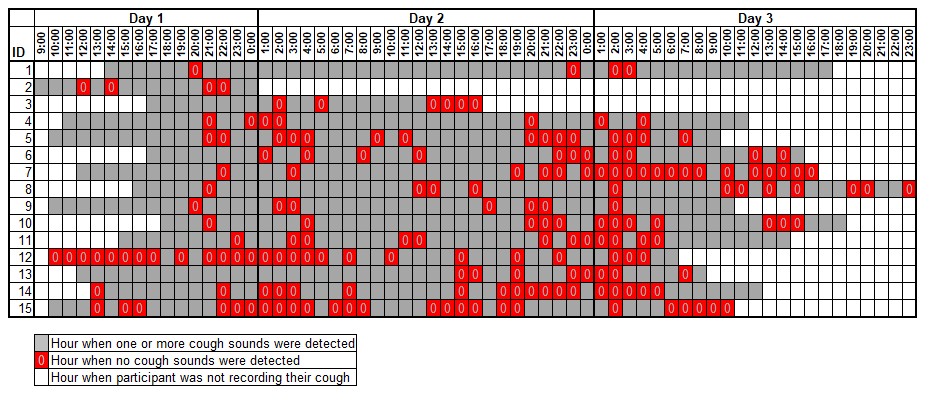

Supplement: S2 Fig — (DOCX) [file pgph.0003530.s002.docx]
