# Supplementary material for: Incidence of cough from acute exposure to fine particulate matter (PM2.5) in Madagascar: A pilot study
Source: PLOS Glob Public Health. 2024 Jul 26;4(7):e0003530. doi: 10.1371/journal.pgph.0003530 (PMC11280240; doi:10.1371/journal.pgph.0003530)
Supplement: S1 Table — (DOCX) [file pgph.0003530.s004.docx]

**S1 Table. Stratified analyses by TB status and smoking status for models with 0 hour lag between hourly PM_2.5_ exposure levels and coughs per hour.**

| **Strata** | **N individuals** | **N hours^*^** | **IRR (95% CI)** | **p-value** |
| --- | --- | --- | --- | --- |
| *Smoking status* | | | | |
| Ever smoked | 4 | 193 | 2.21 (1.74, 2.80) | <0.001 |
| Never smoked | 11 | 485 | 1.22 (0.96, 1.55) | 0.107 |
| *TB status* | | | | |
| TB positive | 5 | 257 | 1.24 (0.95, 1.60) | 0.108 |
| TB negative | 10 | 421 | 1.47 (1.11, 1.94) | 0.006 |

N, number; IRR, incidence rate ratio; CI, confidence interval

^*^Number of hours of cough and PM_2.5_ used in the analysis.
