# Supplementary material for: Incidence of cough from acute exposure to fine particulate matter (PM2.5) in Madagascar: A pilot study
Source: PLOS Glob Public Health. 2024 Jul 26;4(7):e0003530. doi: 10.1371/journal.pgph.0003530 (PMC11280240; doi:10.1371/journal.pgph.0003530)
Supplement: Attachment — Submitted filename: 2024-06-05 - Letter - v02.docx [file pgph.0003530.s005.docx]

Journal Requirements:

1. Please review your reference list to ensure that it is complete and correct. If you have cited papers that have been retracted, please include the rationale for doing so in the manuscript text, or remove these references and replace them with relevant current references. Any changes to the reference list should be mentioned in the rebuttal letter that accompanies your revised manuscript. If you need to cite a retracted article, indicate the article’s retracted status in the References list and also include a citation and full reference for the retraction notice.

There are no retracted references in the reference list. We have added two new references:

- Ref. 28 on the effect of smoking on cough to justify the stratified analyses presented in the supplementary according to smoking status.
- Ref 30 showing that cough frequency tends to decrease in response to anti-TB treatment.

2. We noticed that you used "data not presented" in the manuscript. We do not allow these references, as the PLOS data access policy requires that all data be either published with the manuscript or made available in a publicly accessible database. Please amend the supplementary material to include the referenced data or remove the references.

Data is now publicly available and referenced on line 142-143. Statement about “data not presented” has been removed (line 150).

3. We have noticed that you have uploaded Supporting Information files, but you have not included a list of legends. Please add a full list of legends for your Supporting Information files after the references list.

We have provided a list of Supporting Information titles after the references. According to the PLOS GPH instructions, legends are optional for Supporting information (<https://journals.plos.org/globalpublichealth/s/supporting-information>).

Reviewer #1: The study is Novel Pilot study. This study can be described as a methods and feasibility study where the details of the process of measuring longitudinal simultaneous PM exposure and its acute effects (cough) are provided. Due to the small sample size, the strength of the association between PM and Cough is not scientifically valid. In addition, including two TB positive cases, smokers can be avoided as the sample size is very small. Hence can analyze the data by excluding the TB and smokers. If available more number of never smokers and non TB participants can be included.

Thank you for your review. We agree that the small sample size makes it difficult to draw definite conclusions on the association between PM2.5 and cough, as has been detailed in our discussion (line 299-301). We were cautious not to include time-invariant characteristics in the main analysis as there was insufficient power with a sample size of n=15 to assess the associations and potential confounding effect these variables may have had. Rather, we focused on providing an overview of the association between time-varying predictor PM2.5 and longitudinally collected cough data.

Removing TB positive and smoking participants would further restrict the sample size, which is not desired. It is also impossible to collect additional participants, as suggested by the reviewer. However, we have considered the potential modification / interactive effect of TB and smoking on the association between PM2.5 and cough frequency by performing stratified analyses, which are now presented in the supplementary section.

Reviewer #2: The research described in the manuscript demonstrates methodological rigor by employing objective measures of PM2.5 exposure and cough frequency monitoring. The study design, including the use of univariable Generalized Estimating Equation (GEE) models, appears technically sound for investigating the association between PM2.5 exposure and respiratory symptoms. The conclusions drawn from the data presented are supported by the findings, which show a statistically significant association between PM2.5 exposure and cough frequency among study participants. Additionally, the manuscript acknowledges potential limitations, such as sample size constraints and confounding variables, and provides suggestions for future research to address these limitations. Overall, the manuscript meets the publication criteria of PLOS Global Public Health by presenting methodologically and ethically rigorous research with conclusions appropriately drawn based on the data presented.

Thank you for your review.

Based on the provided author statement, the authors have made the code for cough screening publicly available on GitHub, which aligns with the requirement for data availability. However, the statement also mentions that the audio source file and PM 2.5 measurements datapoints are available upon request to the corresponding author, rather than being fully accessible without restriction. While this approach may be acceptable in some cases, it does not fully comply with the PLOS Data policy, which typically requires that all data underlying the findings described in the manuscript be made fully available without restriction, with rare exceptions. Therefore, the manuscript may not fully meet the data availability requirements of PLOS Global Public Health. To ensure compliance with the journal's publication criteria, the authors should consider providing a more comprehensive Data Availability Statement, specifying any restrictions on publicly sharing the data.

Thank you for this, we have now made the full cough, PM2.5 and clinical/demographic predictor dataset publicly available through Synapse.org. The exact reference is on line 142-143.

Additional changes to manuscript: We have slightly modified the start/end recording times of some participants due to a slight discrepancy that was overlooked upon initial submission. This change does not affect the interpretation of results nor the main conclusions drawn. Changes are presented in track change and affect the distribution of PM2.5 for participants (Table 1) and the associations (Table 2). The changes in association are minor, with shifts in the IRR estimates by the second decimal place.
